# Structural Studies of Lipopolysaccharide-defective Mutants from *Brucella melitensis* Identify a Core Oligosaccharide Critical in Virulence*

**DOI:** 10.1074/jbc.M115.701540

**Published:** 2016-02-11

**Authors:** Carolina Fontana, Raquel Conde-Álvarez, Jonas Ståhle, Otto Holst, Maite Iriarte, Yun Zhao, Vilma Arce-Gorvel, Seán Hanniffy, Jean-Pierre Gorvel, Ignacio Moriyón, Göran Widmalm

**Affiliations:** From the ‡ Department of Organic Chemistry, Arrhenius Laboratory, Stockholm University, S-106 91 Stockholm, Sweden,; the § Instituto de Salud Tropical, Instituto de Investigación Sanitaria de Navarra, and Departamento de Microbiología y Parasitología, Universidad de Navarra, c/Irunlarrea 1, 31008 Pamplona, Spain,; the ¶ Division of Structural Biochemistry, Leibniz-Center for Medicine and Biosciences, Priority Area Asthma and Allergy, Research Center Borstel, Airway Research Center North, Member of the German Center for Lung Research, D-23845 Borstel, Germany,; the ‖ Centre d'Immunologie de Marseille-Luminy Aix-Marseille University, UM2 Marseille, France,; ** INSERM, U1104 Marseille, France, and; ‡‡ CNRS, UMR7280 Marseille, France

**Keywords:** glycosyltransferase, Gram-negative bacteria, lipopolysaccharide (LPS), mutant, nuclear magnetic resonance (NMR), Brucella melitensis, WadC

## Abstract

The structures of the lipooligosaccharides from *Brucella melitensis* mutants affected in the WbkD and ManB_core_ proteins have been fully characterized using NMR spectroscopy. The results revealed that disruption of *wbkD* gives rise to a rough lipopolysaccharide (R-LPS) with a complete core structure (β-d-Glc*p*-(1→4)-α-Kdo*p*-(2→4)[β-d-Glc*p*N-(1→6)-β-d-Glc*p*N-(1→4)[β-d-Glc*p*N-(1→6)]-β-d-Glc*p*N-(1→3)-α-d-Man*p*-(1→5)]-α-Kdo*p*-(2→6)-β-d-Glc*p*N3N4*P*-(1→6)-α-d-Glc*p*N3N1*P*), in addition to components lacking one of the terminal β-d-Glc*p*N and/or the β-d-Glc*p* residues (48 and 17%, respectively). These structures were identical to those of the R-LPS from *B. melitensis* EP, a strain simultaneously expressing both smooth and R-LPS, also studied herein. In contrast, disruption of *manB_core_* gives rise to a deep-rough pentasaccharide core (β-d-Glc*p*-(1→4)-α-Kdo*p*-(2→4)-α-Kdo*p*-(2→6)-β-d-Glc*p*N3N4*P*-(1→6)-α-d-Glc*p*N3N1*P*) as the major component (63%), as well as a minor tetrasaccharide component lacking the terminal β-d-Glc*p* residue (37%). These results are in agreement with the predicted functions of the WbkD (glycosyltransferase involved in the biosynthesis of the O-antigen) and ManB_core_ proteins (phosphomannomutase involved in the biosynthesis of a mannosyl precursor needed for the biosynthesis of the core and O-antigen). We also report that deletion of *B. melitensis wadC* removes the core oligosaccharide branch not linked to the O-antigen causing an increase in overall negative charge of the remaining LPS inner section. This is in agreement with the mannosyltransferase role predicted for WadC and the lack of Glc*p*N residues in the defective core oligosaccharide. Despite carrying the O-antigen essential in *B. melitensis* virulence, the core deficiency in the *wadC* mutant structure resulted in a more efficient detection by innate immunity and attenuation, proving the role of the β-d-Glc*p*N-(1→6)-β-d-Glc*p*N-(1→4)[β-d-Glc*p*N-(1→6)]-β-d-Glc*p*N-(1→3)-α-d-Man*p*-(1→5) structure in virulence.

## Introduction

*Brucella* is a genus of Gram-negative facultative intracellular coccobacilli that causes brucellosis in humans and animals. Although its true extent is not known and the disease is largely unreported (1), it has been estimated that there are half a million new cases every year, most of them located in the poorest rural areas of the world (2). Humans can acquire brucellosis by ingestion of unpasteurized milk from infected animals or by contact with their secretions, but generally they are not themselves a source of contagion. The species within this genus were originally differentiated on the basis of their primary host preferences, with *Brucella melitensis* (sheep and goat), *Brucella suis* (pig), and *Brucella abortus* (cattle) being the most common in domestic livestock. Epidemiological evidence shows that, among them, *B. melitensis* is the most virulent species for humans (3).

**FIGURE 1.**
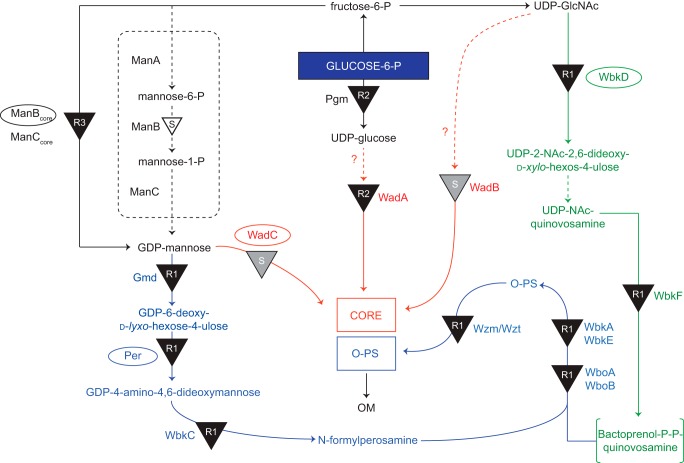
**Proposed pathways involved in the biosynthesis of the S-LPS of *B. melitensis*.** Overview of the pathways involved in the biosynthesis of the S-LPS of *B. melitensis* (adapted from Ref. 10). The steps leading to *N*-formylperosamine synthesis and to its polymerization are indicated in *blue*, and those leading to bactoprenol priming for *N*-formylperosamine polymerization are depicted in *green*. Subsequently, the O-PS is translocated to the periplasm by the Wzm/Wzt ABC transporter (also in *blue*) and ligated to the core oligosaccharide that results from the pathways marked in *red*. The LPS phenotypes obtained by disrupting the different steps (indicated by *black triangles*) are annotated (*R1*, *R2*, or *R3*). A *white-filled triangle* (GDP-mannose pathway) indicates a mutation that does not generate an R phenotype, and *gray triangles* marked with S (*middle*) indicate a mutation that, while blocking the synthesis of a core lateral branch, does not prevent O-PS linkage to the core. Enzymes disrupted in this study (WadC, Per, WbkD, and ManB_core_) are indicated with an *ellipse*.

The lipopolysaccharides (LPS) of Gram-negative bacteria are exposed on the cell surface, and three different regions with different chemical and biological properties can be identified as follows: the lipid A, the core oligosaccharides, and the polysaccharide, which in most cases represents the O-specific polysaccharide (O-PS,^2^ O-antigen) (4, 5). The LPS of *Brucella* shows very low endotoxicity, which illustrates poor detection by innate immunity. Thus, it is considered one of the virulence factors that allow the pathogen to escape early detection by the host immune system (6, 7). Whereas this characteristic is related to the structure of the lipid A and core oligosaccharide of LPS, the O-PS (8, 9) also plays a major role in virulence, because it has been repeatedly observed that mutants lacking the O-PS (*i.e.* producing a rough (R) type LPS, also termed lipooligosaccharide (LOS)) are attenuated (10). *Brucella* strains with R-LPS are often caused by spontaneous mutations (11–13) and can also be due to mutation in genes encoding proteins involved in the biosynthesis of the monosaccharide components of the O-PS, its polymerization or transport, or the core oligosaccharide. In a previous report, the genes involved in the biosynthesis of the LPS of *B. melitensis* were screened, and several mutants affected in the biosynthesis of both the core oligosaccharide or O-PS were obtained (10). To assign mutations to the biosynthetic pathways, the mutants were classified as R1 (complete core), R2 (defective core), and R3 (deep R core), respectively, according to the decrease in their LPS apparent molecular mass (10). For instance, mutations in *wbkD*, *wadA,* and *manB_core_* gave rise to R1, R2, and R3 LPS core glycoforms, respectively (10). Based on sequence homology comparisons, *wbkD* was proposed to code for a putative epimerase/dehydratase involved in the biosynthesis of quinovosamine, the monosaccharide located at the reducing end of the O-PS (10, 14, 15). In contrast, *wadA* was proposed to encode a glycosyltransferase involved in the biosynthesis of the core, whereas *manB_core_* was proposed to encode a phosphomannomutase involved in the biosynthesis of GDP-mannose, used as a precursor in the synthesis of both the core oligosaccharide and the perosamine residues found in the O-PS (10, 16). Moreover, it was shown in later works that deletion of genes encoding glycosyltransferases WadB and WadC creates severe but uncharacterized defects in the core without affecting the section linked to the O-PS. Remarkably, *B. abortus* w*adC* mutants are attenuated despite carrying an intact O-PS (17). An overview of the proposed pathways involved in the biosynthesis of the smooth LPS of *B. melitensis* is shown in Fig. 1.

In this study, we describe the structural elucidation of the LOS from *B. melitensis* strain EP, a spontaneous mutant producing O-PS and increased amounts of LOS (18), Bm_*wbkD* and Bm_*manB_core_*, two mutants in *wbkD* and *manB_core_*, disrupted in the corresponding biosynthetic steps that produce R1 and R3 LOS, respectively (Table 1 and Fig. 1). We also report that deletion of *B. melitensis wadC* removes the core oligosaccharide section not linked to the O-PS. We furthermore show that, despite carrying the O-PS essential in virulence, the lack of this core section results in a marked change in bacterial surface physicochemical properties, a more efficient detection by innate immunity, and attenuation in both cellular and animal models.

**TABLE 1:** ***B. melitensis* LPS mutants used in the present work**

Name	Parental strain	Gene (ORF)	Role (annotation)	LPS phenotype	Refs.
Bm_*manB_core_*	H38	*manB_core_* (BMEII0899)	Core synthesis (phosphomannomutase )	R3	10
Bm_*wbkD*	H38	*wbkD* (BMEI1427)	O-PS synthesis (epimerase/dehydratase)	R1	10
Bm_*per*	16M	*per* (BMEI1414)	O-PS synthesis (perosamine synthetase)	R1	10, 46, 47
Bm_*wadC*	16M	*wadC* (BMEI0509)	Core synthesis (glycosyltransferase)	S	This work
Bm_*wadC_per*	16M	*wadC* (BMEI0509)/*per* (BMEI1414)	Core and O-PS synthesis	R3	This work

## Experimental Procedures

### 

#### 

##### Bacterial Strains

*B. melitensis* 16M (biovar 1 reference strain) and *B. melitensis* H38 are wild type virulent strains commonly used in virulence studies that are identical with regard to LPS genes (10). *B. melitensis* EP (henceforth BmEPR) is a strain that, although virulent and able to synthesize O-polysaccharides, produces comparatively large proportions of R-LPS (18) and is thus suitable to obtain R-LPS in large amounts for chemical analyses. Mutants with defects in LPS genes (Table 1 and Fig. 1) were obtained, characterized, and stored according to the procedures described previously (10, 17).

##### Growth of Bacteria and Isolation of the LOS

Bacteria were propagated in tryptic soy broth either in a Biostat fermentor (BmEPR, Bm_*wbkD*, and Bm_*manB_core_*) or in 2-liter flasks (Bm_*wadC* and Bm_*wadC_per*) on an orbital shaker in a BSL3 facility. After 36 h of incubation, bacteria were inactivated with phenol, harvested by tangential flow filtration, and washed twice with saline (19). For LOS extraction (BmEPR, Bm_*wbkD*, Bm_*manB_core_*, and Bm_*wadC_per*), bacteria were first acetone-dried and then extracted following the phenol/chloroform/light petroleum method (20), and the overall defect (R1, R2, and R3) in the R-LPS was confirmed by SDS-PAGE and silver staining and absence of reactivity with O-PS specific monoclonal antibodies (10). The LPS of Bm_*wadC* was obtained from the phenol phase of a water/phenol extract and further purified as described before (21).

##### Compositional Analyses

Quantitative analyses of sugars, organic bound phosphate, and fatty acids were performed as described previously (22).

##### Preparation of the Deacylated Oligosaccharides

The LOS from the different *B. melitensis* mutants were *O*-deacylated using hydrazine, followed by *N*-deacylation with hot KOH (23) and purified by high performance anion-exchange chromatography.

##### Mass Spectrometry

Electrospray ionization high resolution mass spectra (ESI-HR-MS) were recorded in negative ion mode using a MicrOTOF^TM^ mass spectrometer (Bruker Daltonics). Nitrogen was used as the collision gas.

##### NMR Spectroscopy

The deacylated LOSs of BmEPR (3.8 mg), Bm_*wbkD* (2.4 mg), and Bm_*manB_core_* (3.4 mg) were deuterium-exchanged by freeze-drying three times from 99.9% D_2_O and examined as a solution of 99.96% D_2_O (0.55 ml) at pD 8 and 25 °C. Chemical shifts are reported in parts/million using internal sodium 3-trimethylsilyl-(2,2,3,3-^2^H_4_)-propanoate (δ_H_ 0.00), external 1,4-dioxane in D_2_O (δ_C_ 67.40), or external 2% phosphoric acid in D_2_O (δ_P_ 0.00) as references.

The diffusion-filtered ^1^H NMR spectrum of the deacylated LOS from Bm_*manB_core_* was recorded on a Bruker Avance 500 MHz spectrometer equipped with a 5-mm Z-gradient (53.0 G·cm^−1^) TCI (^1^H/^13^C/^15^N) CryoProbe using the one-dimensional stimulated spin-echo pulse sequence with bipolar gradients and LED (ledbpgp2s1d) (24). Diffusion-encoded sinusoidal gradients pulses (δ/2) of 1.8 ms and a strength of 70% of the maximum were used; the diffusion time was set to 100 ms. The one-dimensional diffusion-filtered ^1^H NMR spectra of the deacylated LOS from BmEPR and Bm_*wbkD* were recorded on a Bruker Avance III 700 MHz spectrometer equipped with a 5-mm Z-gradient (53.0 G·cm^−1^) TCI (^1^H/^13^C/^15^N) CryoProbe, using the same pulse sequence as described above and diffusion-encoded smoothed square-shape gradient pulses (δ/2) of 1.8 ms and a strength of 50% of the maximum; the diffusion time was set to 120 ms.

^1^H and ^13^C NMR chemical shifts assignments of the three LOS materials referred to in the preceding paragraph were obtained from experiments recorded at a magnetic field strength of 16.4 tesla. ^1^H chemical shift assignments were obtained using ^1^H,^1^H TOCSY experiments (25) employing the States-TPPI method, an MLEV-17 spin-lock of 10 kHz, and different mixing times (between 20 and 100 ms). To identify correlations from possible impurities of lower molecular mass than the deacylated LOSs from Bm_*manB_core_*, a diffusion-filtered ^1^H,^1^H TOCSY experiment (τ*_m_* = 100 ms) was acquired employing the pulse sequence “ledbpgpml2s2d” from the standard Bruker library; diffusion-encoded smoothed square-shape gradients pulses (δ/2) of 1.8 ms and strength of 70% of the maximum were used and a diffusion time of 150 ms.

^13^C NMR chemical shifts were obtained from the respective ^13^C spectra, and the assignments were carried out using multiplicity-edited ^1^H,^13^C HSQC experiments (26) employing the echo/anti-echo method. Adiabatic pulses (27, 28) were used for ^13^C inversion (smoothed CHIRP, 20%, 80 kHz, 500 μs, *Q* = 5.0) (29) and refocusing (composite smoothed CHIRP, 80 kHz, 2.0 ms). The ^1^H,^13^C-H2BC experiments (30) were recorded with a constant-time delay of 33 ms, and the ^1^H,^13^C HSQC TOCSY experiments were acquired using an MLEV-17 spin-lock of 10 kHz employing mixing times ranging from 20 to 100 ms.

For assignments of inter-residue correlations, ^1^H,^1^H NOESY and ^1^H,^13^C heteronuclear multiple-bond correlation (HMBC) experiments were utilized. The gradient-selected ^1^H,^1^H NOESY experiments (31) were recorded with a mixing time of 100 ms, whereas the gradient-selected ^1^H,^13^C HMBC experiments (32) were carried out with an evolution time of 65 ms. Band-selective constant-time ^1^H,^13^C HMBC experiments (33) with 2-fold low pass J-filters (hmbcctetgpl2nd) were also employed to improve spectral resolution in the anomeric region. The experiments were recorded over a spectral region of 5.4 × 9.0 ppm with 2048 × 256 data points, using an 80-ms delay for evolution of the long range couplings. A selective ^13^C excitation pulse (*Q*3 gaussian cascade) of 2.5 ms was applied at the center of the anomeric region.

^31^P-based NMR experiments were obtained on a Bruker Avance III 600 MHz spectrometer equipped with a 5-mm Z-gradient (55.7 G·cm^−1^) inverse TXI (^1^H/^13^C/^31^P) probe. The one-dimensional ^1^H-decoupled ^31^P NMR spectra were recorded with a spectral width of 396 ppm, and the ^31^P chemical shifts assignments were obtained from gradient selected ^1^H,^31^P HMBC (32, 34) and ^1^H,^31^P-hetero TOCSY experiments (35). The ^1^H,^31^P HMBC spectra were recorded with an evolution time of 100 ms. The ^1^H,^31^P-hetero TOCSY experiments were carried out with mixing times of 23 and 46 ms, using a DIPSI2 mixing sequence set at 5 kHz on both channels or with a mixing time of 92 ms using a DIPSI2 mixing sequence set at 2.5 kHz.

Additionally, NMR experiments selected from those described above were carried out on deacylated tetra- and pentasaccharides from Bm_*wadC_per* on 0.28 and 0.26 mg, respectively, in D_2_O (0.55 ml) at pD 7, a temperature of 15 °C, and a ^1^H frequency of 500 MHz.

##### ζ Potential

The surface charge density was measured as the electrophoretically effective potential (ζ potential, ζ_sm_) as described previously (10). For this, bacteria were grown in tryptic soy broth, inactivated with 0.5% phenol, washed, and resuspended in 1 mm CsCl, 10 mm HEPES (pH 7.2) at an *A*_600_ of 0.2. Measurements were performed at 25 °C in a Zetamaster instrument using the PCS 1.27 software (Malvern Instruments Ltd., Malvern, UK).

##### Virulence Assays

Bone marrow cells were isolated from femurs of a 7–8-week-old C57Bl/6 female mice and differentiated into dendritic cells (BMDCs) as described by Inaba *et al.* (36). Infections were performed by centrifuging the bacteria onto the differentiated cells (400 × *g* for 10 min at 4 °C; bacteria/cell ratio of 20:1 followed by incubation at 37 °C for 30 min under a 5% CO_2_ atmosphere). BMDCs were gently washed with medium to remove extracellular bacteria before incubating in a medium supplemented with 100 μg·ml^−1^gentamicin for 1 h to kill extracellular bacteria. Thereafter, the antibiotic concentration was decreased to 20 μg·ml^−1^. To monitor *Brucella* intracellular survival, BMDCs were lysed with 0.1% (v/v) Triton X-100 in H_2_O, and serial dilutions of lysates were rapidly plated onto tryptic soy agar plates to enumerate the colony-forming units (cfu).

Seven-week-old female BALB/c mice (Charles River, Elbeuf, France) were kept in cages with water and food *ad libitum* and accommodated under BSL3 biosafety containment 2 weeks before and during the experiments in the facilities of the “CIMA” (registration code ES31 2010000132). The animal handling and other procedures were in accordance with the current European (directive 86/609/EEC) and Spanish (RD 53/2013) legislations, supervised by the Animal Welfare Committee of the University of Navarra (CEEA 045/12). Inocula were prepared in sterile 10 mm PBS (pH 6.85), and ∼5 × 10^4^ cfu in 0.1 ml (the exact dose was assessed retrospectively by plating dilutions of the inocula) were administered intraperitoneally to each mouse. For each strain, 10 mice were inoculated, and the number of cfu in spleen was determined at 2 and 8 weeks after inoculation as described previously (37). The individual data were normalized by logarithmic transformation.

##### Flow Cytometry

To analyze activation and maturation, BMDCs were analyzed for surface expression of classical maturation markers at 24 h post-treatment with the different LPS variants. Cells were labeled with fluorochrome-conjugated antibodies specific for mouse CD11c:PE-Cy7 (clone N418), IA-IE:PE (MHC class II clone M5/114.15.2) (PE), CD86:FITC (Clone GL-1), CD40:AlexaFluor 647 (clone 3/23), and CD80:PE-Cy5 (clone 16-10A1), all from BioLegend. Labeled cells were then subjected to multicolor cytometry using an LSR II UV spectrophotometer (BD Biosciences), and the data were analyzed using FlowJo software by first gating on the CD11c^+^ population (100,000 events) prior to quantifying expression of receptors. Cells were stimulated with 10 μg·ml^−1^ of LPS purified from the *B. melitensis* wild type or Bm_*wadC* mutant strain or with *Escherichia coli* LPS (O55:B5) as a positive control.

##### Cytokine Measurement

Murine IL-6, IL-12p70, and TNF-α were quantified in culture supernatants of stimulated BMDC by sandwich enzyme-linked immunosorbent assays (ELISA) according to the manufacturer's protocol (eBioscience).

## Results

### Compositional Analyses

The analysis of sugars, fatty acids, and organic bound phosphate (P) of the LOS from mutants Bm_manB*_core_*, Bm_*wbkD*, and BmEPR revealed the presence (in nmol·mg^−1^ LOS) of Glc (164/176/110, respectively), Kdo (714/536/572), GlcN (53/637/680), P (1017/782/659), 12:0(3-OH) (88/96/138), 16:0(3-OH) (316/258/283), 16:0 (349/286/188), 18:0 (100/89/102), cyclo19:0 (55/154/105), and 28:0(27-OH)/28:0(27-oxo) (161/99/90). GlcN3N was not quantified. Mannose was present in trace amounts in the LOS from BmEPR but was lacking in the other two LOSs, because it was not present at all in Bm_manB*_core_* and was not released under the acidic hydrolysis conditions used due to its substitution by GlcN (compare structures below).

### Mass Spectrometry

#### 

##### HRMS of the LOS from Bm_manB_core_

**FIGURE 2.**
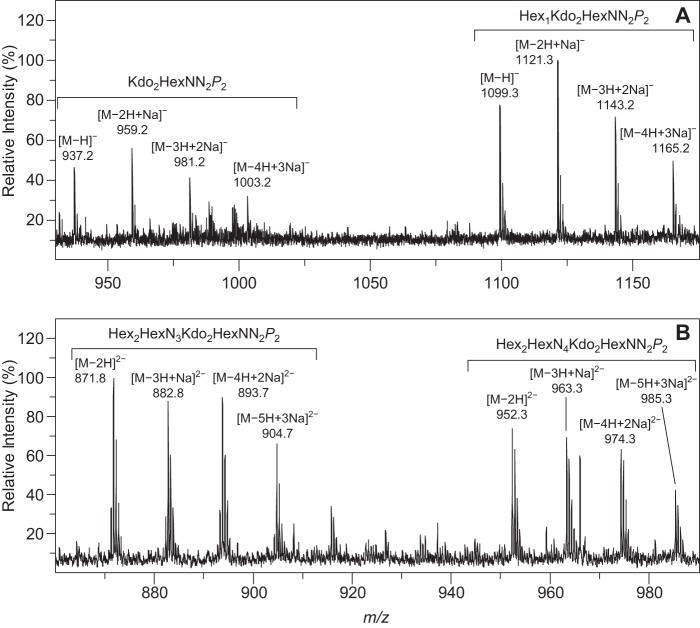
**Mass spectra of deacylated LOS.** Selected regions of the high resolution mass spectra of the deacylated core oligosaccharides of Bm_*manB_core_* (*A*) and Bm_*wbkD* (*B*) recorded in negative ion mode. The clusters of pseudo-molecular ions originating from the two major components of each sample are annotated (see Table 2). Note that the peaks observed in *A* and *B* correspond to singly and doubly negatively charged ion species, respectively.

The two sets of single negatively charged pseudo-molecular ions observed in the high resolution mass spectrum of the deacylated LOS from Bm_*manB_core_* (Fig. 2*A*) are consistent with the presence of two different oligosaccharides. The ions at *m/z* 1099.3 and 937.2 correspond to species formed through the loss of a proton. The former ion is in agreement with the presence of a pentasaccharide composed of one hexose (Hex), two Kdo, two diaminohexoses (HexNN), and two phosphate groups (P), whereas the latter indicate the presence of a tetrasaccharide composed of two Kdo residues, two diaminohexoses, and two phosphate groups (see Table 2). Different singly negatively charged sodium adducts of the pentasaccharide are observed at *m/z* 1121.3, 1143.2, and 1165.2, whereas those of the tetrasaccharide can be found at *m/z* 959.2, 981.2, and 1003.2.

**TABLE 2:** **HR-MS data (negative ion mode) and proposed compositions of the LPS from Bm_*manB_core_* and Bm_*wbkD*** Summary of the diagnostic pseudo-molecular ions observed in the high resolution mass spectra of the deacylated LOS from Bm_*manB_core_* and Bm_*wbkD* and the proposed composition of the two main components present in each sample. The components are abbreviated as follow: Hex (hexoses), HexN (hexosamine), HexNN (diaminohexose), and P (phosphate group).

Mutant name	Pseudo-molecular ions (*m/z*)				Proposed composition (exact molecular mass)
	Observed	Calculated	Annotation	Molecular formula	
Bm_*manB_core_* (Fig. 2*A*)	1099.2680	1099.2750	[M − H]^−^	C_34_H_61_N_4_O_32_P_2_^−^	Hex_1_Kdo_2_HexNN_2_*P*_2_ (1100.2822)
	1121.2520	1121.2569	[M − 2H + Na]^−^	C_34_H_60_N_4_O_32_P_2_Na^−^	
	1143.2344	1143.2389	[M − 3H + 2Na]^−^	C_34_H_59_N_4_O_32_P_2_Na_2_^−^	
	1165.2195	1165.2208	[M − 4H + 3Na]^−^	C_34_H_58_N_4_O_32_P_2_Na_3_^−^	
	937.2173	937.2221	[M − H]^−^	C_28_H_51_N_4_O_27_P_2_^−^	Kdo_2_HexNN_2_*P*_2_ (938.2294)
	959.1989	959.2041	[M − 2H + Na]^−^	C_28_H_50_N_4_O_27_P_2_Na^−^	
	981.1896	981.1860	[M − 3H + 2Na]^−^	C_28_H_49_N_4_O_27_P_2_Na_2_^−^	
	1003.1579	1003.1680	[M − 4H + 3Na]^−^	C_28_H_48_N_4_O_27_P_2_Na_3_^−^	
Bm_*wbkD* (Fig. 2*B*)	952.2962	952.2978	[M − 2H]^2−^	C_64_H_114_N_8_O_53_P_2_^2 −^	Hex_2_HexN_4_Kdo_2_HexNN_2_*P*_2_ (1906.6103)
	963.2803	963.2888	[M − 3H + Na]^2−^	C_64_H_113_N_8_O_53_P_2_Na^2−^	
	974.2724	974.2798	[M − 4H + 2Na]^2−^	C_64_H_112_N_8_O_53_P_2_Na_2_^2−^	
	985.2674	985.2708	[M − 5H + 3Na]^2−^	C_64_H_111_N_8_O_53_P_2_Na_3_^2−^	
	871.7581	871.7635	[M − 2H]^2−^	C_58_H_103_N_7_O_49_P_2_^2−^	Hex_2_HexN_3_Kdo_2_HexNN_2_*P*_2_ (1745.5415)
	882.7532	882.7544	[M − 3H + Na]^2−^	C_58_H_102_N_7_O_49_P_2_Na^2−^	
	893.7469	893.7454	[M − 4H + 2Na]^2−^	C_58_H_101_N_7_O_49_P_2_Na_2_^2−^	
	904.7361	904.7364	[M − 5H + 3Na]^2−^	C_58_H_100_N_7_O_49_P_2_Na_3_^2−^	

##### HRMS of the LOS from Bm_wbkD

The mass spectrum of the deacylated core oligosaccharide from Bm_*wbkD* (Fig. 2*B*) is also consistent with the presence of two major oligosaccharides, which produce the doubly negatively charged ions at *m/z* 952.3 and 871.8 through the loss of two protons each. The ion with higher *m/z* value corresponds to a decasaccharide composed of two Hex, four hexosamines (HexN), two Kdo, two HexNN, and two phosphate groups (see Table 2); the different doubly charged sodium adducts of this oligosaccharide appear at *m/z* 963.3, 974.3, and 985.3. The ion at *m/z* 871.8 corresponds to a nonasaccharide composed of two Hex, three HexN, two Kdo, two HexNN, and two phosphate groups; ions from three different sodium adducts of this compound are present at *m/z* 882.8, 893.7, and 904.7.

### NMR Spectroscopy

#### 

##### LOS from Bm_manB_core_

**FIGURE 3.**
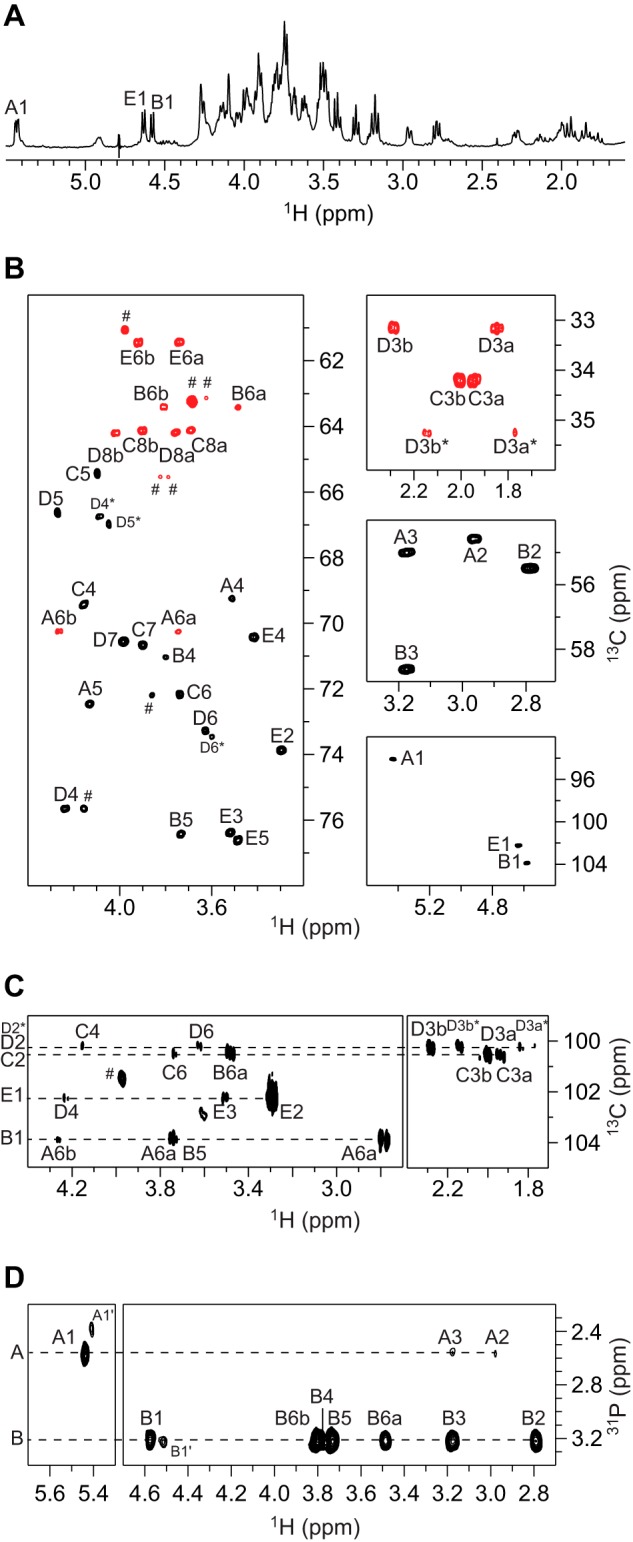
**NMR spectra of the deacylated LOS from Bm_*manB_core_*.** *A,* selected region of the diffusion-filtered ^1^H NMR spectrum. *B,* selected regions of the multiplicity-edited ^1^H,^13^C HSQC spectrum showing the anomeric region (*right bottom*), the region for the nitrogen-bearing carbons (*right middle*), the region for the 3-deoxy-groups of the Kdo residues (*right top*), and the region for the ring atoms and those from hydroxymethyl groups (*left*) in which the cross-peaks from the latter appear in *red. C,* selected region of the ^1^H,^13^C HMBC spectrum showing intra- and inter-residue correlations from anomeric carbons. *D,* selected region of the ^1^H,^31^P-hetero TOCSY spectrum (τ*_m_* = 92 ms) showing correlations from the phosphate groups in residues **A** and **B**. Signals from impurities of lower molecular mass than the LOS oligosaccharides of *B. melitensis* are indicated by the *hash symbol*.

**FIGURE 4.**
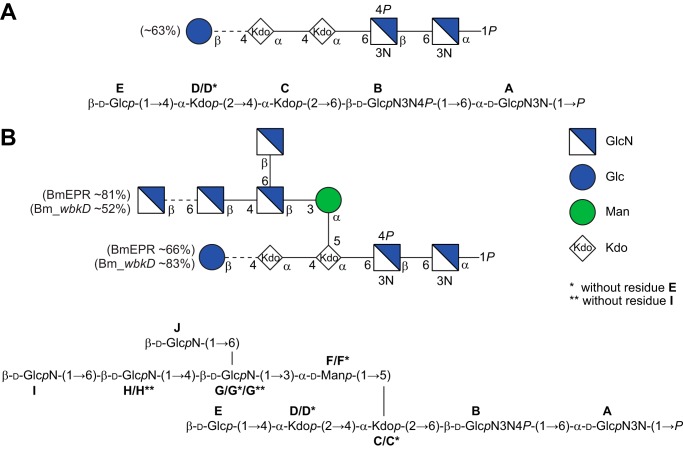
**Structures of the deacylated LOS from Bm_*manB_core_*, Bm_*wbkD,* and BmEPR.** *A,* structure of the penta- and tetra-saccharides in CFG format (*top*) and standard nomenclature (*bottom*) obtained by deacylation of the LOS from Bm_*manB_core_. B,* structure of the deca-, nona-, and octasaccharides in CFG format (*top*) and standard nomenclature (*bottom*) obtained by deacylation of the LOS from BmEPR and Bm_*wbkD*. The ratio of the different oligosaccharides present in the samples was estimated by integration of selected cross-peaks in the multiplicity-edited ^1^H,^13^C HSQC spectrum; the **D3b** and D3b* resonances were used to estimate the ratio of oligosaccharides with and without residue **E**, whereas the **G4** and G4** resonances were used to estimate the ratio of oligosaccharides with and without residue **I**.

In the ^1^H NMR spectrum, three resonances corresponding to anomeric protons were identified as follows: two doublets at 4.577 and 4.632 ppm (^3^*J*_H1,H2_ = 8.1 and 8.0 Hz, respectively) and one doublet of doublets at 5.430 ppm (^3^*J*_H1,H2_ = 3.2 Hz and ^3^*J*_P,H1_ = 8.2 Hz). The residues were named **A**, **E**, and **B**, in order of decreasing ^1^H chemical shifts (Fig. 3*A*). Two major resonances were identified in the ^31^P NMR spectrum at 2.6 and 3.2 ppm, suggesting the presence of two phosphomonoester groups, one of which is consistent with the splitting of the H1 resonance of residue **A**. All the protons from H1 to H6 could be traced using ^1^H,^1^H TOCSY experiments, indicating that these residues have the *gluco*-configuration. ^13^C NMR chemical shifts were assigned using multiplicity-edited ^1^H,^13^C HSQC (Fig. 3*B*) and ^1^H,^13^C H2BC experiments. Both C2 and C3 resonances of residues **A** and **B** were found in the region between 54 and 59 ppm (Fig. 3*B, right middle*), which indicates that these are nitrogen-bearing carbons. ^1^*J*_C1,H1_ couplings constants were extracted from a coupled ^1^H,^13^C HSQC spectrum and revealed that residues **B** and **E** are β-linked (^1^*J*_C1,H1_ = 164 and 163 Hz, respectively), whereas residue **A** is α-linked (^1^*J*_C1,H1_ = 174 Hz); thus **A** is α-d-Glc*p*N3N, **B** is β-d-Glc*p*N3N, and residue **E** is β-d-Glc*p*. In the ^13^C NMR spectrum, three anomeric carbons were identified at 100.10, 100.15, and 100.46 ppm and attributed to C2 resonances in three different populations of Kdo residues (D*, **D,** and **C**, respectively). In the ^1^H,^13^C HMBC spectrum, the C2 carbon of each Kdo residue could be correlated to their respective H3 protons via two-bond heteronuclear correlations (Fig. 3*C*), and the H3 resonances were used as starting points for the assignments of the respective spin systems. The multiplicity-edited ^1^H,^13^C HSQC spectrum (Fig. 3*B*, *right top*) showed cross-peaks of different relative intensities for each of the Kdo residues, which was confirmed by integration of the H3a resonances in the ^1^H NMR spectrum (1.0 H, 0.6 H, and 0.4 H, in residues **C**, **D**, and D*, respectively) and indicated the presence of two different oligosaccharide components. ^1^H and ^13^C chemical shifts assignments are compiled in Table 3. The α-configuration of the anomeric center of the Kdo residues was established by comparison of the ^13^C chemical shifts of the D*-C moiety with those of the same moiety found in a tetrasaccharide with similar structure, previously reported in the literature (38). Inter-residue correlations were observed in the ^1^H,^13^C HMBC spectrum from all the anomeric carbons to the respective protons at the substitution positions (Fig. 3*C*), as well as from the anomeric protons (in residues **E** and **B**) to the respective glycosyloxylated carbons in the next residue (Table 3). These results are in agreement with a major (∼63%) pentasaccharide component (**E**-**D**-**C**-**B**-**A**) in which a Kdo residue (**D**) is 4-substituted with a Glc residue (**E**) and a minor (∼37%) tetrasaccharide component (D*-**C**-**B**-**A**) in which the Glc residue is absent, and consequently the corresponding Kdo residue (D*) is non-substituted. The percentages of each component were determined by integration of the C3/H3b cross-peaks of residues **D** and D* in the ^1^H,^13^C HSQC spectrum. The substitution positions of the phosphomonoester groups were determined using ^1^H,^31^P HMBC and ^1^H,^31^P-hetero TOCSY experiments and were found at C1 in residue **A** (^3^*J*_P,H1_ = 8.2 Hz, ^2^*J*_P,C1_ = 5.4 Hz, and ^3^*J*_P,C2_ = 6.1 Hz) and C4 in residue **B** (Fig. 3*D*). Three additional resonances of minor intensities were found in the ^31^P NMR spectrum at δ_P_ 4.0 (t, ^3^*J*_P,H_ = 7.0 Hz), 4.1 (d, ^3^*J*_P,H_ = 8.2 Hz), and 4.4 (t, ^3^*J*_P,H_ = 6.8 Hz) and were attributed to free phosphoethanolamine, glycerol 2-phosphate, and glycerol 3-phosphate, respectively. The ^1^H resonances of these three components could readily be identified in the ^1^H,^31^P HMBC and ^1^H,^31^P-hetero TOCSY spectra and correlated to their respective carbons in the multiplicity-edited ^1^H,^13^C HSQC (denoted with the *hash symbol* in Fig. 3*B, left*). The chemical shifts assignments of these three components (phosphoethanolamine, 3.975/61.05 and 3.210/41.48 ppm; glycerol 2-phosphate, 4.155/75.64 and 3.682/62.24 ppm; glycerol 3-phosphate, 3.858/72.19, 3.822/65.52, 3.787/65.52, 3.686/63.11, and 3.616/63.11 ppm) were in agreement with data previously reported in the literature (39, 40). Diffusion-filtered experiments (^1^H NMR and ^1^H,^1^H TOCSY spectra) were employed to confirm that these compounds had lower masses than the deacylated LOS material of Bm_*manB_core_* and thus were not linked to LPS. The genome of *B. melitensis* contains an LptA homologue putatively involved in the transference of ethanolamine to an unknown position of the core-lipid A, which could account for the 2-aminoethyl phosphate group (16). This may have been substituting the O4 position of residue **B** (either as phosphodiester or diphosphodiester), but under the basic conditions used to prepare the oligosaccharides, this was probably lost (41–43). Additional spin systems of lower intensity, similar to **A** and **B** but with slightly different chemical shifts, were also identified in the ^1^H,^1^H TOCSY spectra (**A′**: 5.406, 2.848, 3.106, 3.480, and 4.093, and **B′**: 4.516, 2.762, 3.152, 3.830, and 3.737, from H1 to H5, respectively), as well as resonances in the ^1^H NMR at 1.286 (CH_2_) and 0.869 (CH_3_) ppm, attributed to residual acyl groups. The structure of the penta- and tetrasaccharide components of the LOS from Bm_*manB_core_* are shown in Fig. 4*A* and are consistent with the structure of the lipid A reported previously for *B. abortus* (44).

**TABLE 3:** **NMR chemical shift assignments of the LOS from Bm_*manB_core_* and Bm_*wadC_per* and inter-residue correlations from ^1^H,^13^C HMBC NMR spectra** The ^1^H, ^13^C, and ^31^P NMR chemical shifts (ppm) of the LOS from Bm_*manB_core_* were determined at 25 °C and pD 8, whereas the ^1^H and ^13^C NMR chemical shifts of the tetra- and pentasaccharide from Bm_*wadC_per* (OS1 and OS2, respectively) were obtained at 15 °C and pD 7. ^3^*J*_H1,H2_ values are given in Hz in parentheses and ^1^*J*_C1,H1_ values in braces. For residue **A,** ^3^*J*_P,H1_ = 8.2 Hz, ^2^*J*_P,C1_ = 5.4 Hz, and ^3^*J*_P,C2_ = 6.1 Hz. ND means not determined.

Sample/sugar residues		^1^H/^13^C								^31^P	HMBC correlations (from anomeric atom)
		1	2	3	4	5	6	7	8		
Bm_*manB_core_*											
→6)-α-d-Glc*p*N3N1*P*	**A**	5.430 (3.2)	2.955	3.171	3.508	4.128	3.742, 4.263			2.6	
		94.08 {174}	54.59	54.97	69.27	72.43	70.23				
→6)-β-d-Glc*p*N3N4*P*-(1→	**B**	4.577 (8.1)	2.786	3.172	3.797	3.730	3.481, 3.804			3.2	C6, **A**
		103.87 {164}	55.47	58.60	71.05	76.41	63.40				H6a/H6b, **A**
→4)-α-Kdo*p*-(2→	**C**			1.940, 2.002	4.156	4.096	3.738	3.897	3.689, 3.899		
		175.75	100.46	34.20	69.45	65.42	72.15	70.65	64.11		H6a, **B**
→4)-α-Kdo*p*-(2→	**D**			1.846, 2.286	4.237	4.270	3.626	3.983	3.754, 4.016		
		177.40	100.15	33.13	75.63	66.60	73.25	70.55	64.16		H4, **C**
α-Kdo*p*-(2→ (without residue E)	D*			1.769, 2.144	4.086	4.047	3.598	3.983	∼3.754, ∼4.016		
		176.66	100.10	35.22	66.71	66.95	73.44	70.57	64.22		H4, C* ^(^*^a^*^)^
β-d-Glc*p*-(1→	**E**	4.632 (8.0)	3.296	3.521	3.418	3.485	3.737, 3.918				C4, **D**
		102.23 {163}	73.84	76.36	70.39	76.59	61.40				H4, **D**

**Bm_*wadC_per* OS1**											
→6)-α-d-Glc*p*N3N1*P*	**A**	5.445	3.031	3.242	3.562	4.143	3.752, 4.268				
		94.05	54.22	55.14	68.88	72.55	70.25				
→6)-β-d-Glc*p*N3N4*P*-(1→	**B**	4.600	2.812	3.219	3.815	3.739	3.491, 3.810				
		104.00	55.40	58.66	70.82	76.47	63.46				
→4)-α-Kdo*p*-(2→	**C**			1.946, 1.987	4.164	4.100	3.743	3.901	3.696, 3.902		
		ND	ND	34.28	69.47	65.54	72.26	70.72	64.17		
α-Kdo*p*-(2→	**D**			1.768, 2.149	4.090	4.050	3.600	3.989	3.760, 4.021		
		ND	100.31	35.16	66.89	67.06	73.54	70.68	64.29		

**Bm_*wadC_per* OS2**											
→6)-α-d-Glc*p*N3N1*P*	**A**	5.448	3.038	3.249	3.572	4.143	3.751, 4.267				
		93.95	54.18	55.02	68.66	72.43	70.15				
→6)-β-d-Glc*p*N3N4*P*-(1→	**B**	4.600	2.811	3.219	3.818	3.744	3.489, 3.809				
		103.82	55.32	58.54	70.69	76.41	63.34				
→4)-α-Kdo*p*-(2→	**C**			1.948, 1.993	4.175	4.101	3.741	3.901	3.695, 3.900		
		ND	100.50	34.24	69.49	65.45	72.22	70.58	64.04		
→4)-α-Kdo*p*-(2→	**D**			1.848, 2.290	4.241	4.277	3.635	3.987	3.757, 4.018		
		ND	100.36	33.05	75.71	66.59	73.30	70.49	64.16		
β-d-Glc*p*-(1→	**E**	4.636	3.292	3.515	3.408	3.485	3.746, 3.921				C4, **D**
		102.31	73.83	76.44	70.35	76.63	61.40				

*^a^* δ_H4_ of residue C* ∼4.156.

##### LOS from BmEPR

**FIGURE 5.**
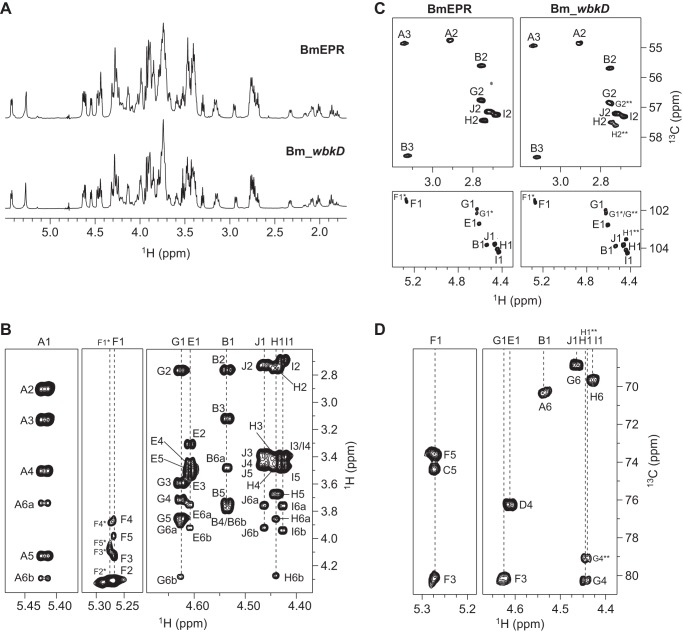
**NMR spectra of the deacylated LOS from Bm_*wbkD* and BmEPR.** *A,* comparison of the diffusion-filtered ^1^H NMR spectra of the deacylated LOS from BmEPR and Bm_*wbkD* (*top* and *bottom*, respectively). *B,* selected regions of the ^1^H,^1^H TOCSY (τ*_m_* = 100 ms) of deacylated LOS from BmEPR showing correlations from anomeric protons. *C,* comparison of selected regions of the multiplicity-edited ^1^H,^13^C HSQC spectrum of the deacylated LOS from BmEPR and Bm_*wbkD* (*left* and *right*, respectively), showing part of the anomeric region (*bottom*) and the region for the nitrogen-bearing carbons (*top*). *D,* selected regions of the ^1^H,^13^C HMBC spectrum of the deacylated LOS from Bm_*wbkD* showing intra- and inter-residue correlations from anomeric protons.

The ^1^H NMR spectrum of the deacylated LOS (Fig. 5*A, top*) revealed a complex material, with several signals of different intensities in the anomeric region (between 5.42 and 4.43 ppm). Eight distinctive spin systems originating from the anomeric protons were identified in the ^1^H,^1^H TOCSY spectra, and the different sugar residues were denoted **A**, **F**, **G**, **E**, **B**, **J**, **H,** and **I**, in order of decreasing ^1^H chemical shifts. In addition, a minor spin system similar to residue **F** but with slightly different chemical shifts was also identified and denoted F*. For residues **A**, **G**, **E**, **B**, **J**, **H,** and **I**, all protons from H1 to H6 could be identified in the ^1^H,^1^H TOCSY spectrum recorded with the longest mixing time (Fig. 5*B*), indicating that these monosaccharide components have the *gluco*-configuration. The distinctive downfield chemical shift of H2 in residues **F** and F* suggested that these are Man residues. The anomeric proton of residue **A** is a doublet of doublets (^3^*J*_H1,H2_ = 3.2 Hz and ^3^*J*_P,H1_ = 8.3 Hz) indicating that this could be the α-d-GlcN3N1*P* residue at the reducing end of the lipid **A** moiety. ^13^C chemical shifts were assigned using multiplicity-edited ^1^H,^13^C HSQC (Fig. 5*C, left*), ^1^H,^13^C-H2BC, ^1^H,^13^C HMBC, and ^1^H,^13^C HSQC TOCSY experiments. ^1^H and ^13^C chemical shifts assignments are compiled in Table 4. Both C2 and C3 resonances of residues **A** and **B** were found in the region between 54 and 59 ppm, confirming that these are the two GlcN3N residues of the lipid A. The C2 resonances of residues **G**, **J**, **H**, and **I** were also found in the region of the nitrogen-bearing carbons, indicating that these are GlcN residues (Fig. 5*C, left top*); thus, residue **E** is Glc. ^1^*J*_C1,H1_ couplings constants were extracted from a coupled ^1^H,^13^C HSQC spectrum and revealed that residues **A** and **F** are α-linked (^1^*J*_C1,H1_ = 173–174 Hz), whereas residues **B, E** and **G-J** are β-linked (^1^*J*_C1,H1_ = 162–165 Hz). The spin systems of the Kdo residues were analyzed as described before, and four distinctive populations were found and denoted **D** and **C** (major populations) and D* and C* (minor populations). Inter-residue correlations from anomeric carbons and protons were extracted from both regular and band-selective ^1^H,^13^C HMBC spectra and used for determination of the sequence of sugar residues in the LOS (Table 4). The same two basic structures as for the LOS from Bm_*manB_core_* could be identified (*i.e.* **E**-**D**-**C**-**B**-**A** and D*-**C**-**B**-**A**), with the only difference being a branched oligosaccharide moiety composed of GlcN residues (**I**-**H**-**[J]G**), in the case of the major component that extends from a Man residue (**F**) linked to position 5 of the Kdo residue **C**. Thus, the major decasaccharide and nonasaccharide components of the LOS (∼66 and ∼34%, respectively, determined by integration of the characteristic C3/H3b cross-peaks of residues **D** and D* in the ^1^H,^13^C HSQC spectrum) have the following sequence of sugar residues: **E**-**D**-[**I**-**H-[J]G**-**F]C**-**B**-**A** and D*-[**I**-**H**-[J]G*-F*]C*-**B**-**A**. The substitution positions of the phosphomonoester groups were studied as described above. The two major resonances in the ^31^P NMR spectrum (2.5 and 3.1 ppm) were attributed to α-d-Glc*p*N3N1*P* (residue **A**) and α-d-Glc*p*N3N4*P* (residue **B**); likewise, the minor resonances found at δ_P_ 4.0, 4.1, and 4.4 were attributed to free ethanolamine phosphate, glycerol 2-phosphate, and glycerol 3-phosphate. Additional cross-peaks of lower intensity (∼19%) were also found in the multiplicity edited ^1^H,^13^C HSQC spectrum and attributed to an oligosaccharide similar to the decasaccharide described above, but without the GlcN residue **I** linked to residue **H**. In this case, ^1^H and ^13^C chemical shift assignments were carried out by comparison with the chemical shifts of the same oligosaccharide found in the LOS from Bm_*wbkD* (∼48%) (see below), and the corresponding sugar residues were denoted with a double asterisk. The C4/H4 cross-peaks of residues **G** and G** in the ^1^H,^13^C HSQC spectrum were used to estimate the percentage of these oligosaccharides in the sample. The structures of the deca- and nonasaccharide components of the LOS from BmEPR are shown in Fig. 4*B*.

**TABLE 4:** **NMR chemical shifts assignments of the resonances of the LOS from BmEPR and Bm_*wbkD*** ^1^H, ^13^C, and ^31^P NMR chemical shifts (ppm) at 25 °C and pD 8 of the resonances of the components of the deacylated LOS from BmEPR and Bm_*wbkD* and inter-residue correlations from the ^1^H,^13^C HMBC spectra. The different oligosaccharide components of the samples differ in the presence/absence of residues **E** (Glc) and/or **I** (GlcN) (see Fig. 4). For residue **A,** ^3^*J*_P,H1_ = 8.3 Hz, ^2^*J*_P,C1_ = ∼4.5 Hz, and ^3^*J*_P,C2_ = ∼7.4 Hz. Resonances from H1 and C1 in residue G* are found at 4.626 and 102.12 ppm, respectively (overlapping with H1 and C1 in residue G**). Resonances from H3 and C3 in F** are found at 4.129 and 80.07 ppm (overlapping with H3 and C3 in residue **F**). ND means not determined. NR means not resolved.

Sugar residues		^1^H/^13^C												^31^P	HMBC correlations (from anomeric atom)
		1		2	3a	3b	4	5	6a	6b	7	8a	8b		
→6)-α-d-Glc*p*N3N1*P*	**A**	5.419	(3.2)	2.898	3.126		3.503	4.127	3.738	4.290				2.5	
		94.28	{174}	54.76	54.87		69.25	72.48	70.34						
→6)-β-d-Glc*p*N3N4*P*-(1→	**B**	4.535	(8.0)	2.753	3.112		3.764	3.724	3.472	3.782				3.1	C6, **A**
		103.81	{162}	55.61	58.63		71.44	76.39	63.66						H6a, **A**
→4,5)-α-Kdo*p*-(2→	**C**				2.010	2.081	4.250	4.236	3.721*^a^*		3.738*^a^*	3.717	3.890		
		175.68*^a^*		100.45	35.25		70.54	74.36	72.80*^a^*		70.46*^a^*	64.27			H6a, **B**
→4)-α-Kdo*p*-(2→ (without residue E)	C*				1.996	2.099	4.177	4.254	ND		ND	ND	ND		
		ND		100.45	35.25		74.96	74.36	ND		ND	ND			
→4)-α-Kdo*p*-(2→	**D**				1.863	2.324	4.205	4.276	3.721		3.987	3.793	3.996		
		175.34*^a^*		101.18	33.25		76.22	66.68	72.83		70.76	63.95			H4, **C**
α-Kdo*p*-(2→ (without residue E)	D*				1.802	2.161	4.042	4.022	3.702		3.994	3.782	3.983		
		ND		101.74	35.29		66.77	67.37	72.96		71.03	64.85			
β-d-Glc*p*-(1→	**E**	4.609	(7.9)	3.299	3.520		3.426	3.480	3.747	3.915					C4, **D**
		102.69	{162}	73.86	76.30		70.30	76.53	61.36						H4, **D**
→3)-α-d-Man*p*-(1→	**F**	5.268	(NR)	4.312	4.129		3.847	4.130	∼3.887						C5, **C**
		101.54	{173}	68.71	80.07		65.79	73.62	61.66						H5, **C**
→3)-α-d-Man*p*-(1→ (without residue E)	F*	5.275		4.310	4.089		3.874	4.089	∼3.887						
		101.40	{173}	68.90	80.47		65.66	73.41	61.66						H5, C*
→4,6)-β-d-Glc*p*N-(1→	**G**	4.627	(8.2)	2.758	3.584		3.709	3.844	3.891	4.272					C3, **F**
		101.91	{165}	56.77	75.20		80.26	74.25	68.89						H3, **F**
→4,6)-β-d-Glc*p*N-(1→ (without residue I)	G**	4.626	(8.30)	2.756	3.595		3.736	3.844	3.891	4.272					
		102.12	{165}	56.87	74.90		79.12	74.25	68.89						H3, F**
→6)-β-d-Glc*p*N-(1→	**H**	4.442	(8.1)	2.748	3.405		3.467	3.668	3.851	4.267					C4, **G**
		104.03	{163}	57.45	76.01		70.48	75.70	69.66						H4, **G**
β-d-Glc*p*N-(1→ (without residue I)	H**	4.440		2.727	3.405		3.480	3.489	3.752	3.934					C4, G**
		103.48	{163}	57.59	76.31		70.41	77.03	61.46						H4, G**
β-d-Glc*p*N-(1→	**I**	4.426	(8.1)	2.682	3.384		3.464	3.460	3.752	3.934					C6, **H**
		104.18	{163}	57.26	76.33*^b^*		70.55	76.87	61.62						H6a, **H**
β-d-Glc*p*N-(1→	**J**	4.465	(8.2)	2.723	3.373		3.409	3.460	3.748	3.913					C6, **G**
		103.77	{162}	57.16	76.30*^b^*		70.55	76.87	61.57						

*^a^* Tentative assignments are shown.

*^b^* Assignments may be interchanged.

##### LOS from Bm_wbkD

The ^1^H NMR spectrum of the deacylated LOS from Bm_*wbkD* (Fig. 5*A, bottom*) appears quite similar to that of BmEPR (Fig. 5*A, top*), with the only difference being the relative intensities displayed by some of the resonances. ^1^H and ^13^C chemical shifts assignments were carried out as described previously; the same oligosaccharide structures as above were identified, **E**-**D**-[**I**-**H**-**[J]G**-**F]C**-**B**-**A** and D*-[**I**-**H**-[J]G*-F*]C*-**B**-**A**, and particularly the nonasaccharide **E**-**D**-[H**-[J]G**-F**]C-**B**-**A**, that was fully characterized due to the higher relative concentration of the component in the sample (∼48%, instead of 19% observed in the case of BmEPR). Comparison of the multiplicity-edited ^1^H,^13^C HSQC spectrum of the deacylated LOS from BmEPR (Fig. 5*C, bottom left*) and that of Bm_*wbkD* (Fig. 5*C, bottom right*) allowed for the identification of a conspicuous resonance at δ_H_/δ_C_ 4.440/103.48 (H1/C1 in residue H**) that is noticeably stronger in the latter spectrum. The ^1^H and ^13^C resonances of residue H** were assigned employing ^1^H,^1^H TOCSY, ^1^H,^13^C HSQC TOCSY, and ^1^H,^13^C HMBC experiments. The H6a and H6b resonances (3.752 and 3.934 ppm, respectively) were identified in the ^1^H,^1^H TOCSY spectrum (τ*_m_* 100 ms) due to their significant chemical shifts differences with respect to those of H6a and H6b in residue **H** and were correlated to the C6 carbon in the multiplicity-edited ^1^H,^13^C HSQC spectrum. The differences in the chemical shifts of C6 in residues H** and **H** (61.46 and 69.66 ppm, respectively) indicated that the former is not 6-substituted and thus is a terminal residue. Another conspicuous signal was observed in the multiplicity-edited ^1^H,^13^C HSQC spectrum at δ_H_/δ_C_ 3.736/79.12 (H4/C4 in residue G**); the ^1^H resonances in that spin system were identified using correlations from the carbon at 79.12 ppm in the ^1^H,^13^C HSQC TOCSY spectrum (τ*_m_* 100 ms), and the corresponding ^13^C resonances were assigned using a multiplicity-edited ^1^H,^13^C HSQC spectrum. Furthermore, an inter-residue correlation was observed in the band-selective constant-time ^1^H,^13^C HMBC spectrum (recorded with enhanced resolution in the carbon anomeric region) between the anomeric carbon of residue H** and the proton at 3.736 ppm (H4 in residue G**). In addition, the regular ^1^H,^13^C HMBC spectrum showed a correlation between the anomeric proton of residue H** and the C4 carbon in residue G** (Fig. 5*D*), thus confirming that the G** residue is 4-substituted with residue H**. Furthermore, the anomeric carbon of residue G** showed an inter-residue correlation in the band-selective constant-time ^1^H,^13^C HMBC spectrum to a proton at 4.129 ppm, attributed to H3 of residue F** (overlapping with H3 of residue **F**). ^1^H and ^13^C chemical shifts assignments of the deca- and nonasaccharide components of the LOS from Bm_*wbkD* are compiled in Table 4; the corresponding structures and relative percentages are shown in Fig. 4*B*.

##### LOS from Bm_wadC_per

**FIGURE 6.**
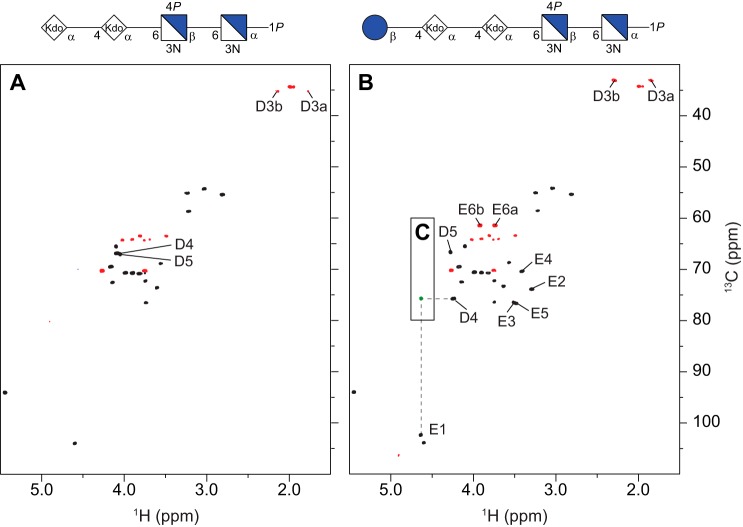
**NMR spectra of the deacylated LOS from Bm_*wadC_per*.** Comparison of the multiplicity-edited ^1^H,^13^C HSQC spectra (*black/red*) of the tetrasaccharide (*A*) and pentasaccharide (*B*) (OS1 and OS2, respectively) isolated from Bm_*wadC_per*; the cross-peaks with significant chemical shift differences between the two oligosaccharides are annotated. The structures (in CFG format) of the respective oligosaccharides are shown on the top of each spectra. *C,* overlay of a selected region of the ^1^H,^13^C HMBC spectrum of the OS2 showing an interglycosidic heteronuclear long range correlation (*green*) between residues **D** and **E**.

Two oligosaccharides were isolated from the deacylated LOS of the double mutant, *viz.* a tetra- and a pentasaccharide (OS1 and OS2, respectively), the structures of which correspond to those described for the oligosaccharide mixture from Bm_*manB_core_*. Their ^1^H and ^13^C NMR chemical shifts (Table 3) were assigned by two-dimensional NMR experiments (*cf.* ^1^H,^13^C HSQC NMR spectra in Fig. 6, *A* and *B*, respectively), in good agreement with those from the Bm_*manB_core_* oligosaccharides. In particular, an interglycosidic heteronuclear correlation was present in the ^1^H,^13^C HMBC spectrum of the pentasaccharide from the anomeric proton (H1) of the glucosyl residue to the glycosyloxylated carbon atom (C4) of the second Kdo residue (Fig. 6*C*), thus establishing and confirming the structural element E-D (Fig. 4).

In a recent investigation of oligo- and polysaccharides obtained by mild acid hydrolysis of LPS from different *Brucella* serotypes, structural elements consistent with core oligosaccharides presented herein were present (15). Notably, it was shown that d-QuiNAc, which is the primer for the O-chain polysaccharide, is β-(1→4)-linked to the glucose residue of the core (*B. suis* data), thereby defining the attachment site of the O-antigen to the core region of *Brucella* serotypes, a finding anticipated to be valid in the *Brucella* serotypes investigated herein.

### Role of Brucella Melitensis Core in Virulence

**FIGURE 7.**
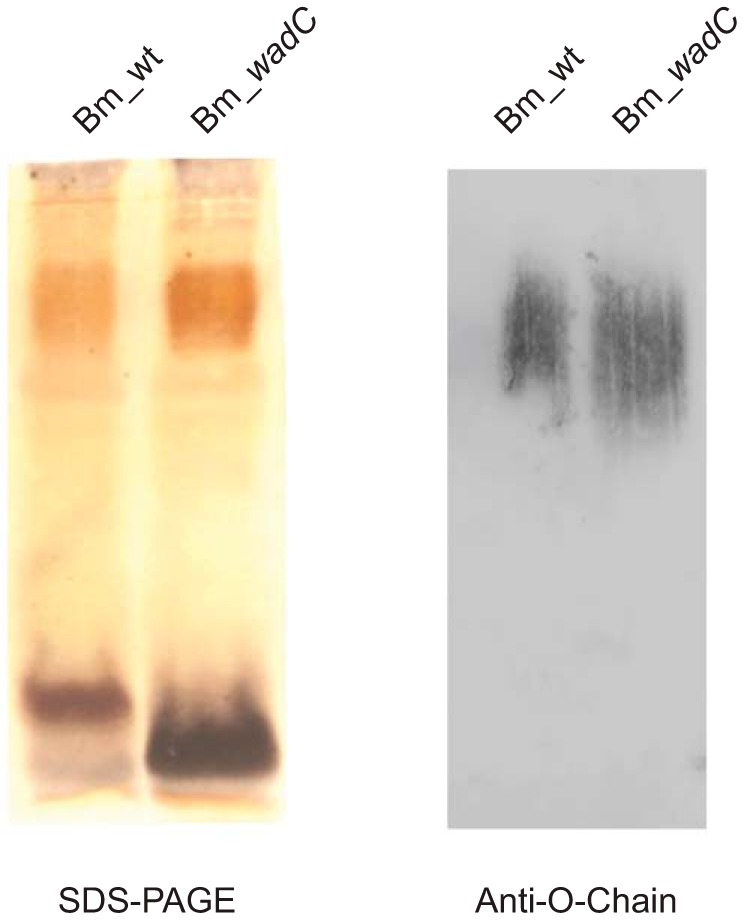
***B. melitensis* WadC mutants carry a core defect that does not affect the linkage to the O-PS.** SDS-PAGE and Western blot analysis with anti-O-chain monoclonal antibody Cby-33H8 of LPS SDS-proteinase K extracts from Bm_wt (*B. melitensis* 16M wild type) and Bm_*wadC (B. melitensis* 16MΔ*wadC* mutant).

**FIGURE 8.**
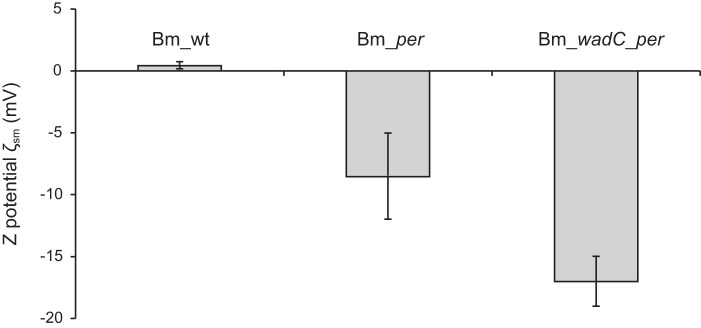
***B. melitensis* core lateral branch shields bacterial surface negatively charged groups (inner core and lipid A).** The figure presents ζ potential measurements of Bm_*per* (no LPS O-PS and complete core oligosaccharide), Bm_*wadC_per* (no LPS O-PS and defective core oligosaccharide) in comparison with *B. melitensis* 16M (wild type strain; Bm_wt). Each *bar* represents the means ± S.E. of 10 measurements of one representative experiment.

For an unambiguous analysis of the role of the LPS core in the virulence of *B. melitensis*, the use of core-defective O-PS-bearing bacteria is necessary. To this end, a mutant in *wadC* was constructed. As shown in Fig. 7, SDS-PAGE and Western blot analyses show that this *wadC* mutant carries a core defect but keeps the O-PS, a result in agreement with previous work in *B. abortus* (17). This phenotype is consistent with the putative role of WadC as a mannosyltransferase because this enzymatic activity would be necessary to create the mannose-Kdo linkage in the structure shown in Fig. 4*B*. To verify this, a double Bm_*wadC_per* mutant was constructed and its LOS analyzed. Consistent with the role of Per in O-PS synthesis (Fig. 1) and the need of the O-PS for export to the periplasm and subsequent linkage to the core oligosaccharide (5), the LOS of this double mutant lacked the full O-PS section (quinovosamine, mannose, and *N*-formyl perosamine polymer). Moreover, this LOS also completely lacked the mannose/glucosamine-containing oligosaccharide linked to Kdo (residue **C**), being similar to that of the *manB_core_* mutant (blocked in mannose synthesis). The lack of aminosugars should increase the negative charge of the inner sections of LPS, and this was shown to occur in ζ_sm_ potential measurements of the Bm_*wadC_per* mutants and Bm_*per* (Fig. 8).

**FIGURE 9.**
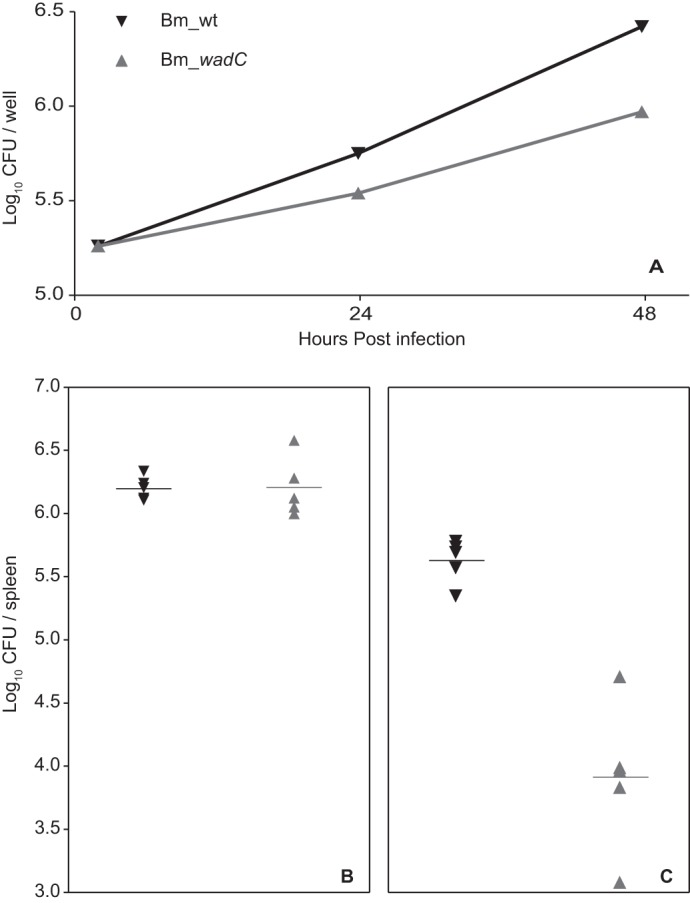
***B. melitensis* mutants defective in the core lateral branch are attenuated in dendritic cells and in mice.** Bone marrow-derived dendritic cells were infected with *B. melitensis* 16M (wild type strain; Bm_wt) or *B. melitensis* 16MΔ*wadC* (Bm_*wadC*), and CFU were measured at the indicated times (each point represents the means ± S.E. of triplicate wells of a representative experiment) (*A*). Groups of five mice were infected with 5 × 10^4^ CFU of Bm_wt (▾) or Bm_*wadC* (▴) and CFU/spleen determined at 2 (*B*) and 8 (*C*) weeks.

To assess the biological effect of the core, the ability of Bm_*wadC* to multiply in BMDCs was studied in comparison with wild type *B. melitensis*. Mutant bacteria displayed a comparatively reduced ability to multiply in these cells suggestive of attenuation (Fig. 9*A*). When the virulence was assessed *in vivo* using the mouse model of brucellosis, Bm_*wadC* and the parental strain yielded similar cfu at post-infection week 2 (Fig. 9*B*). At post-infection week 8, however, the mutant was present in comparatively reduced numbers in the spleens (Fig. 9*C*).

**FIGURE 10.**
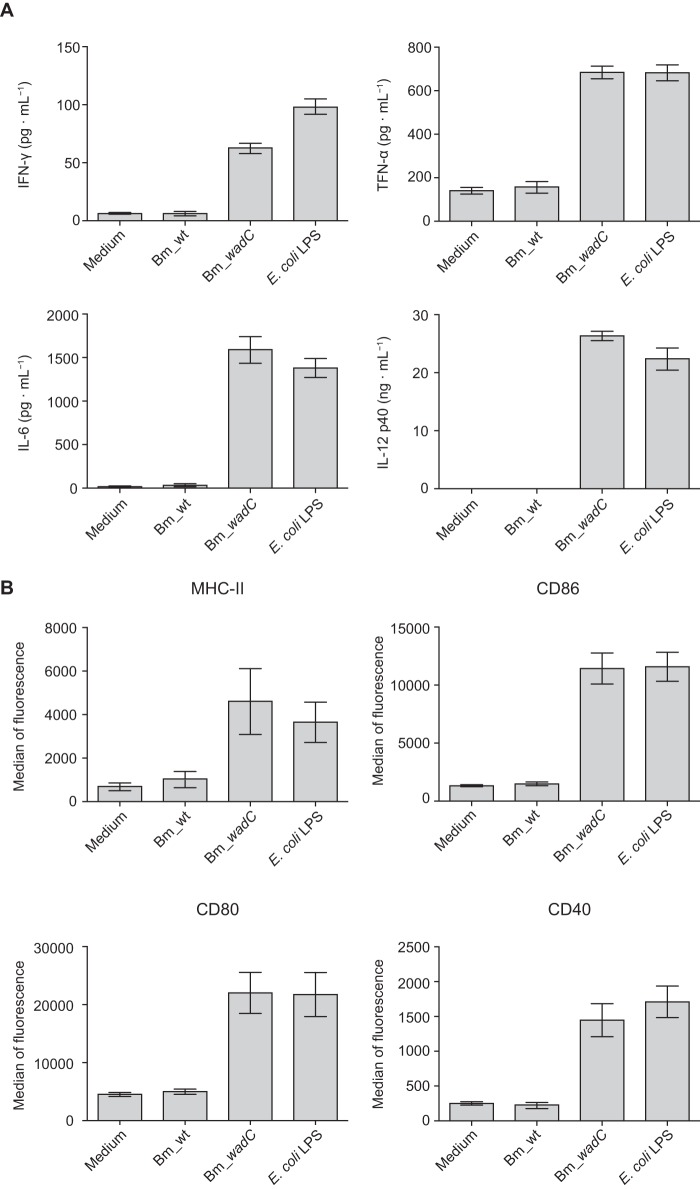
***B. melitensis wadC* LPS triggers dendritic cell activation and maturation.** Mouse BMDCs were stimulated for 24 h with medium or purified LPS from *B. melitensis* 16M (wild type strain; Bm_wt), *B. melitensis* 16MΔwadC (*Bm_wadC*), or *E. coli* (O55:B5), all administered at an equivalent molarity (0.25 μm). IFN-γ, TNF-α, IL-6, and IL-12p40 secretion levels in culture supernatant were determined by ELISA (*A*). Surface levels of MHC-II, CD86, CD80, and CD40 and were measured by flow cytometry (*B*). Data are presented as the means ± S.E. of at least five independent experiments.

To determine whether the reduced virulence of Bm_*wadC* was associated with an alteration in the immunogenicity of its LOS structure, further experiments were performed to assess its potential to induce pro-inflammatory responses in BMDCs. Unlike the Bm_wt LPS, which induced no secretion of inflammatory cytokines, Bm_*wadC* LPS induced the release of the pro-inflammatory cytokines IFN-γ, IL-12p40, IL-6, and TNF-α at high levels that were comparable with those obtained with LPS from *E. coli* (Fig. 10*A*). Similarly, with *E. coli* LPS, Bm_*wadC-*stimulated BMDCs underwent maturation as judged by the surface expression of MHC-II and the co-stimulatory markers CD86, CD80, and CD40. By contrast, BMDCs treated with Bm_wt LPS maintained an immature phenotype with no evident up-regulation of these surface receptors (Fig. 10*B*). Taken together, these results demonstrate that an intact LPS core is not only required for full virulence of *B. melitensis*, but it also contributes to limiting the activation and maturation of dendritic cells while undergoing replication in these target cells. An alteration in the LPS core, as demonstrated here with Bm_*wadC*, would appear to confer a more endotoxigenic phenotype rendering the pathogen more visible to host target cells, attenuating its intracellular replicative capacity and virulence in mice.

## Discussion

Rough mutants of *B. melitensis* have been reported to show attenuated responses with respect to strains having a smooth LPS. In addition, phenotypes with different core defect structures have been observed to give rise to different attenuation patterns, and this has been associated with changes on the bacterial surface (10, 17). Therefore, knowledge of the primary structure of the LOS may help not only to have a better understanding of the genetics involved in biosynthetic processes but also to comprehend the interaction of the bacteria with the host immune system. Herein, we present a comprehensive study of the structures of different LOSs extracted from four *B. melitensis* strains. These include the native R-LPS (LOS) obtained from a strain producing increased proportions of R-LPS (18) (BmEPR), a mutant in which the *wbkD* gene was disrupted (Bm_*wbkD*) and a mutant affected in the ManB_core_ protein (Bm_*manB_core_*) as well as the double mutant strain Bm_*wadC_per*.

The structural elucidation of the deacylated LOSs from BmEPR and Bm_*wbkD*, carried out using NMR spectroscopy, revealed that both mutants are capable of producing a complete core structure (Fig. 4*B*) as well as minor components lacking the terminal residue **I** (19 and 48%, respectively) and/or residue **E** (34 and 17%, respectively). Furthermore, the pseudo-molecular ions observed in the mass spectra of the deacylated LOS from Bm_*wbkD* (Fig. 2*B* and Table 2) were in agreement with the two major components determined by NMR spectroscopy as follows: (i) a complete core decasaccharide with a molecular formula of C_64_H_116_N_8_O_53_P_2_ that is consistent with a structure containing two Hex (Glc and Man), four HexN (GlcN), two Kdo, and two HexNN residues bearing phosphomonoester substituents (GlcN3N1*P* and GlcN3N4*P*), and (ii) a nonasaccharide with a molecular formula of C_58_H_104_N_7_O_49_P_2_ that is consistent with the same components as above but having three GlcN residues instead of four. It needs to be stressed that the full structures obtained for BmEPR and Bm_*wbkD* are identical. *B. melitensis* EP was used in this work because it keeps the ability to synthesize S-LPS while producing increased amounts of R-LPS, which makes possible to obtain larger amounts of a native R-LPS for chemical analysis. Because a complete core structure was present in the LOS from Bm_*wbkD*, the *wbkD* gene can be anticipated to encode for a protein involved in the biosynthesis of the O-PS. This is in agreement with the predicted function of WbkD as involved in the biosynthesis of QuiNAc, the undecaprenol-priming residue and therefore the first sugar of the O-PS chain (Fig. 1) (10). In a smooth LPS the O-PS chain, having a 3-substituted QuiNAc residue located at its reducing end, would be extended from the O4 position of the Glc residue (15). Consequently, the pentasaccharide moiety composed of GlcN residues linked to a mannosyl residue constitutes a core branch.

The mutant in the *manB_core_* gene showed a deeply truncated core (Fig. 4*A*) in which the lateral branch is absent. This is in agreement with previous observations that such a kind of mutants (Bm_*manB_core_*) have a lower molecular mass LOS (R3 phenotype) than those of BmEPR and Bm_*wbkD* (R1 phenotype) and fail to react with monoclonal antibodies specific for the outer core (10, 16). The studies carried out using NMR spectroscopy showed that the LOS from Bm_*manB_core_* is composed of two major oligosaccharide components as follows: a pentasaccharide and a tetrasaccharide (63 and 37%, respectively), with the only difference being the presence or absence of the terminal Glc residue, respectively (Fig. 4*A*). These structures are in agreement with the two sets of pseudo-molecular ions observed in the mass spectrum that correspond to structures of general formula C_34_H_62_N_4_O_32_P_2_ and C_28_H_52_N_4_O_27_P_2_ (Fig. 2*A* and Table 2), respectively. The ManB_core_ has been previously predicted to be the phosphomutase involved in the biosynthesis of GDP-mannose (Fig. 1), which is used in the biosynthesis of monosaccharide components of the core and O-PS. Indeed, it is shown here that when the *manB_core_* gene is blocked the LOS oligosaccharide lacks the mannosyl residue and the branched structure that extends from it.

From a biological point of view, the structure described here is not a trivial one as it explains a number of characteristics related to the virulence of an important pathogen. The brucellae are resistant to components of the innate immune system such as bactericidal peptides and complement while prompting a weak pro-inflammatory response during infection (7), characteristics that are in part related to the biological and physicochemical properties of its LPS (6, 7, 17). Many of these properties have been attributed to the poor cytokine-inducing characteristics of *Brucella* lipid A, on account of its very long chain fatty acids that may prevent effective recognition by TLR4-MD2 (6). However, it has been predicted that the core should play a complementary role whereby its structure allows it to conceal the inner sections of lipid A, making them less accessible for binding by the TLR4-MD2 complex or for binding bactericidal peptides or complement (15, 17). The loss in monoclonal antibody reactivity observed with a *B. abortus wadC* mutant suggested that the core of smooth brucellae may atypically be composed of two separate oligosaccharide branches, one linking the lipid A to the O-PS and another that protrudes laterally thereby concealing the negatively charged groups essential for interaction with the TLR4-MD2 complex (17). The characterization presented here confirms these predictions as the GlcN-rich oligosaccharide stemming from the first Kdo residue (**C**) also creates a positively charged structure that can neutralize the negatively charged groups of Kdo and lipid A, the primary targets of innate immunity receptors and effector molecules. This is further supported by the attenuated intracellular replicative/virulence profile and change in surface charge exhibited by the Bm_*wadC* mutant strain, as well as the enhanced ability of its LPS to stimulate maturation and pro-inflammatory cytokine secretion (including Th1-type cytokines IL-12 and IFN-γ) when compared with its WT counterpart.

In addition to reaffirming the above hypothesis, these observations provide new understanding of the important role of *Brucella* core-LPS in influencing key host-pathogen interactions during the early stages of infection and perhaps when chronic disease develops. Moreover, our findings have important implications as they identify *Brucella* LPS as a key target/protagonist for strategies aimed toward the development of safer attenuated brucellosis vaccines that can promote more efficient protective Th1 responses (45), thought to be critical for the control of intracellular brucellae.

## Author Contributions

I. M. and G. W. conceived and coordinated the study. O. H., J. P. G., I. M., and G. W. supervised the study. C. F., R. C. A., J. S., M. I., Y. Z., V. A. G. and S. H. performed experiments and genomic analyses. C. F., R. C. A., J. S., I. M., and G. W. wrote the manuscript. All authors analyzed the results and approved the final version of the manuscript.

## Footnotes

2 The abbreviations used are:

O-PS: O-specific polysaccharide
LOS: lipooligosaccharide
R-LPS: rough lipopolysaccharide
BMDC: bone marrow-derived dendritic cell
PE: phycoerythrin
Kdo: 3-deoxy-d-*manno*-oct-2-ulosonic acid
HSQC: heteronuclear single quantum coherence
HMBC: heteronuclear multiple-bond correlation
HR-MS: high resolution mass spectrometry.

## References

[1] Dean A. S., Crump L., Greter H., Schelling E., and Zinsstag J. (2012) Global burden of human brucellosis: a systematic review of disease frequency. PLoS Negl. Trop. Dis. 6, e1865 23145195 10.1371/journal.pntd.0001865 PMC3493380

[2] McDermott J., Grace D., and Zinsstag J. (2013) Economics of brucellosis impact and control in low-income countries. Rev. Sci. Tech. 32, 249–261 23837382 10.20506/rst.32.1.2197

[3] Ariza J. (1999) Brucellosis: an update. The perspective from the Mediterranean basin. Rev. Med. Micriobiol. 10, 125–135

[4] Raetz C. R., and Whitfield C. (2002) Lipopolysaccharide endotoxins. Annu. Rev. Biochem. 71, 635–700 12045108 10.1146/annurev.biochem.71.110601.135414 PMC2569852

[5] Valvano M. A., Furlong S. E., and Patel K. B. (2011) in Bacterial Lipopolysaccharides (Knirel Y. A., and Valvano M. A., eds) pp. 275–310, Springer-Verlag, Wien

[6] Lapaque N., Moriyon I., Moreno E., and Gorvel J.-P. (2005) *Brucella* lipopolysaccharide acts as a virulence factor. Curr. Opin. Microbiol. 8, 60–66 15694858 10.1016/j.mib.2004.12.003

[7] Barquero-Calvo E., Chaves-Olarte E., Weiss D. S., Guzmán-Verri C., Chacón-Díaz C., Rucavado A., Moriyón I., and Moreno E. (2007) *Brucella abortus* uses a stealthy strategy to avoid activation of the innate immune system during the onset of infection. PLoS ONE 2, e631 17637846 10.1371/journal.pone.0000631 PMC1910614

[8] Knirel Y. A. (2011) in Bacterial Lipopolysaccharides. Structure, Chemical Synthesis, Biogenesis and Interaction with Host Cells (Knirel Y. A., and Valvano M. A., eds) pp. 41–115, Springer, Vienna

[9] Zaccheus M. V., Ali T., Cloeckaert A., Zygmunt M. S., Weintraub A., Iriarte M., Moriyón I., and Widmalm G. (2013) The epitopic and structural characterization of *Brucella suis* biovar 2 O-polysaccharide demonstrates the existence of a new M-negative C-negative smooth *Brucella serovar*. PLoS ONE 8, e53941 23335981 10.1371/journal.pone.0053941 PMC3545991

[10] González D., Grilló M.-J., De Miguel M.-J., Ali T., Arce-Gorvel V., Delrue R.-M., Conde-Alvarez R., Muñoz P., López-Goñi I., Iriarte M., Marín C.-M., Weintraub A., Widmalm G., Zygmunt M., Letesson J.-J., et al (2008) Brucellosis vaccines: assessment of *Brucella melitensis* lipopolysaccharide rough mutants defective in core and O-polysaccharide synthesis and export. PLoS ONE 3, e2760 18648644 10.1371/journal.pone.0002760 PMC2453230

[11] Turse J. E., Pei J., and Ficht T. A. (2011) Lipopolysaccharide-deficient *Brucella* variants arise spontaneously during infection. Front. Microbiol. 2, 54 21833310 10.3389/fmicb.2011.00054 PMC3153030

[12] Mancilla M., López-Goñi I., Moriyón I., and Zárraga A. M. (2010) Genomic island 2 is an unstable genetic element contributing to *Brucella* lipopolysaccharide spontaneous smooth-to-rough dissociation. J. Bacteriol. 192, 6346–6351 20952568 10.1128/JB.00838-10 PMC3008527

[13] Mancilla M., Marín C. M., Blasco J. M., Zárraga A. M., López-Goñi I., and Moriyón I. (2012) Spontaneous excision of the O-polysaccharide *wbkA* glycosyltransferase gene is a cause of dissociation of smooth to rough *Brucella* colonies. J. Bacteriol. 194, 1860–1867 22328663 10.1128/JB.06561-11 PMC3318470

[14] Zygmunt M. S., Blasco J. M., Letesson J.-J., Cloeckaert A., and Moriyón I. (2009) DNA polymorphism analysis of *Brucella* lipopolysaccharide genes reveals marked differences in O-polysaccharide biosynthetic genes between smooth and rough *Brucella* species and novel species-specific markers. BMC Microbiol. 9, 92 19439075 10.1186/1471-2180-9-92 PMC2698832

[15] Kubler-Kielb J., and Vinogradov E. (2013) The study of the core part and non-repeating elements of the O-antigen of *Brucella* lipopolysaccharide. Carbohydr. Res. 366, 33–37 23261780 10.1016/j.carres.2012.11.004 PMC3540177

[16] Iriarte M., González D., Delrue R. M., Monreal D., Conde-Álvarez R., López-Goñi I., Letesson J.-J., and Moriyón I. (2004) in Brucella: Molecular and Cellular Biology (López-Goñi I., and Moriyón I., eds) pp. 159–192, Horizon Scientific Press Ltd., Norfolk, UK

[17] Conde-Álvarez R., Arce-Gorvel V., Iriarte M., Manček-Keber M., Barquero-Calvo E., Palacios-Chaves L., Chacón-Díaz C., Chaves-Olarte E., Martirosyan A., von Bargen K., Grilló M.-J., Jerala R., Brandenburg K., Llobet E., Bengoechea J. A., et al (2012) The lipopolysaccharide core of *Brucella abortus* acts as a shield against innate immunity recognition. PLoS Pathog. 8, e1002675 22589715 10.1371/journal.ppat.1002675 PMC3349745

[18] Bowden R. A., Verger J. M., Grayon M., Limet J. N., and Dubray G. (1993) Simultaneous expression of smooth and rough phase properties related to lipopolysaccharide in a strain of *Brucella melitensis*. J. Med. Microbiol. 39, 363–370 8246253 10.1099/00222615-39-5-363

[19] Aragón V., Díaz R., Moreno E., and Moriyón I. (1996) Characterization of *Brucella abortus* and *Brucella melitensis* native haptens as outer membrane O-type polysaccharides independent from the smooth lipopolysaccharide. J. Bacteriol. 178, 1070–1079 8576040 10.1128/jb.178.4.1070-1079.1996 PMC177767

[20] Galanos C., Lüderitz O., and Westphal O. (1969) A new method for the extraction of R-lipopolysaccharides. Eur. J. Biochem. 9, 245–249 5804498 10.1111/j.1432-1033.1969.tb00601.x

[21] Velasco J., Bengoechea J. A., Brandenburg K., Lindner B., Seydel U., González D., Zähringer U., Moreno E., and Moriyón I. (2000) *Brucella abortus* and its closest phylogenetic relative, *Ochrobactrum* spp., differ in outer membrane permeability and cationic peptide resistance. Infect. Immun. 68, 3210–3218 10816465 10.1128/iai.68.6.3210-3218.2000 PMC97564

[22] Posch G., Andrukhov O., Vinogradov E., Lindner B., Messner P., Holst O., and Schäffer C. (2013) Structure and immunogenicity of the rough-type lipopolysaccharide from the periodontal pathogen *Tannerella forsythia*. Clin. Vaccine Immunol. 20, 945–953 23616409 10.1128/CVI.00139-13 PMC3675976

[23] Holst O., Thomas-Oates J. E., and Brade H. (1994) Preparation and structural analysis of oligosaccharide monophosphates obtained from the lipopolysaccharide of recombinant strains of *Salmonella minnesota* and *Escherichia coli* expressing the genus-specific epitope of *Chlamydia lipopolysaccharide*. Eur. J. Biochem. 222, 183–194 7515346 10.1111/j.1432-1033.1994.tb18856.x

[24] Wu D., Chen A., and Johnson C. S. (1995) An improved diffusion-ordered spectroscopy experiment incorporating bipolar-gradient pulses. J. Magn. Reson. Ser. A 115, 260–264

[25] Bax A., and Davis D. G. (1985) MLEV-17-based two-dimensional homonuclear magnetization transfer spectroscopy. J. Magn. Reson. 65, 355–360

[26] Schleucher J., Schwendinger M., Sattler M., Schmidt P., Schedletzky O., Glaser S. J., Sørensen O. W., and Griesinger C. (1994) A general enhancement scheme in heteronuclear multidimensional NMR employing pulsed field gradients. J. Biomol. NMR 4, 301–306 8019138 10.1007/BF00175254

[27] Tannús A., and Garwood M. (1997) Adiabatic pulses. NMR Biomed. 10, 423–434 9542739 10.1002/(sici)1099-1492(199712)10:8<423::aid-nbm488>3.0.co;2-x

[28] Kupě Ē. (2001) Applications of adiabatic pulses in biomolecular nuclear magnetic resonance. Methods Enzymol. 338, 82–111 11460562 10.1016/s0076-6879(02)38216-8

[29] Böhlen J.-M., and Bodenhausen G. (1993) Experimental aspects of Chirp NMR spectroscopy. J. Magn. Reson. Ser. A 102, 293–301

[30] Nyberg N. T., Duus J. Ø., and Sørensen O. W. (2005) Heteronuclear two-bond correlation: suppressing heteronuclear three-bond or higher NMR correlations while enhancing two-bond correlations even for vanishing ^2^*J*_CH_. J. Am. Chem. Soc. 127, 6154–6155 15853304 10.1021/ja050878w

[31] Kumar A., Ernst R. R., and Wüthrich K. (1980) A two-dimensional nuclear Overhauser enhancement (2D NOE) experiment for the elucidation of complete proton-proton cross-relaxation networks in biological macromolecules. Biochem. Biophys. Res. Commun. 95, 1–6 7417242 10.1016/0006-291x(80)90695-6

[32] Bax A., and Summers M. F. (1986) ^1^H and ^13^C assignments from sensitivity-enhanced detection of heteronuclear multiple-bond connectivity by 2D multiple quantum NMR. J. Am. Chem. Soc. 108, 2093–2094

[33] Claridge T. D., and Pérez-Victoria I. (2003) Enhanced ^13^C resolution in semi-selective HMBC: a band-selective, constant-time HMBC for complex structure elucidation by NMR. Org. Biomol. Chem. 1, 3632–3634 14649890 10.1039/b307122g

[34] Zartler E. R., and Martin G. E. (2011) The use of ^1^H-^31^P GHMBC and covariance NMR to unambiguously determine phosphate ester linkages in complex polysaccharide mixtures. J. Biomol. NMR 51, 357–367 21922277 10.1007/s10858-011-9563-8

[35] Kellogg G. W. (1992) Proton-detected hetero TOCSY experiments with application to nucleic acids. J. Magn. Reson. 98, 176–182

[36] Inaba K., Inaba M., Romani N., Aya H., Deguchi M., Ikehara S., Muramatsu S., and Steinman R. M. (1992) Generation of large numbers of dendritic cells from mouse bone marrow cultures supplemented with granulocyte/macrophage colony-stimulating factor. J. Exp. Med. 176, 1693–1702 1460426 10.1084/jem.176.6.1693 PMC2119469

[37] Grilló M.-J., Blasco J. M., Gorvel J. P., Moriyón I., and Moreno E. (2012) What have we learned from brucellosis in the mouse model? Vet. Res. 43, 29 22500859 10.1186/1297-9716-43-29 PMC3410789

[38] Holst O., Broer W., Thomas-Oates J. E., Mamat U., and Brade H. (1993) Structural analysis of two oligosaccharide bisphosphates isolated from the lipopolysaccharide of a recombinant strain of *Escherichia coli* F515 (Re chemotype) expressing the genus-specific epitope of *Chlamydia* lipopolysaccharide. Eur. J. Biochem. 214, 703–710 7686488 10.1111/j.1432-1033.1993.tb17971.x

[39] De Boer W. R., Kruyssen F. J., and Wouters J. T. (1976) The structure of teichoic acid from *Bacillus subtilis* var. *niger* WM as determined by ^13^C nuclear-magnetic-resonance spectroscopy. Eur. J. Biochem. 62, 1–6 815085 10.1111/j.1432-1033.1976.tb10090.x

[40] Holst O., Röhrscheidt-Andrzejewski E., and Brade H. (1990) Isolation and characterisation of 3-deoxy-d-*manno*-2-octulopyranosonate 7-(2-aminoethyl phosphate) from the inner core region of *Escherichia coli* K-12 and *Salmonella minnesota* lipopolysaccharides. Carbohydr. Res. 204, 93–102 2279250 10.1016/0008-6215(90)84024-o

[41] Mühlradt P. F., Wray V., and Lehmann V. (1977) A ^31^P nuclear magnetic resonance study of the phosphate groups in lipopolysaccharide and lipid A from *Salmonella*. Eur. J. Biochem. 81, 193–203 590267 10.1111/j.1432-1033.1977.tb11941.x

[42] Stewart A., Bernlind C., Martin A., Oscarson S., Richards J. C., and Schweda E. K. H. (1998) Studies of alkaline mediated phosphate migration in synthetic phosphoethanolamine l-glycero-d-*manno*-heptoside derivatives. Carbohydr. Res. 313, 193–202

[43] Brabetz W., Müller-Loennies S., Holst O., and Brade H. (1997) Deletion of the heptosyltransferase genes *rfaC* and *rfaF* in *Escherichia coli* K-12 results in an Re-type lipopolysaccharide with a high degree of 2-aminoethanol phosphate substitution. Eur. J. Biochem. 247, 716–724 9266718 10.1111/j.1432-1033.1997.00716.x

[44] Qureshi N., Takayama K., Seydel U., Wang R., Cotter R. J., Agrawal P. K., Bush C. A., Kurtz R., and Berman D. T. (1994) Structural analysis of the lipid A derived from the lipopolysaccharide of *Brucella abortus*. J. Endotoxin Res. 1, 137–148

[45] Conde-Álvarez R., Arce-Gorvel V., Gil-Ramírez Y., Iriarte M., Grilló M.-J., Gorvel J. P., and Moriyón I. (2013) Lipopolysaccharide as a target for brucellosis vaccine design. Microb. Pathog. 58, 29–34 23219811 10.1016/j.micpath.2012.11.011

[46] Godfroid F., Taminiau B., Danese I., Denoel P., Tibor A., Weynants V., Cloeckaert A., Godfroid J., and Letesson J. J. (1998) Identification of the perosamine synthetase gene of *Brucella melitensis* 16M and involvement of lipopolysaccharide O side chain in *Brucella Survival in Mice and in Macrophages*. Infect. Immun. 66, 5485–5493 9784561 10.1128/iai.66.11.5485-5493.1998 PMC108687

[47] Zhao G., Liu J., Liu X., Chen M., Zhang H., and Wang P. G. (2007) Cloning and characterization of GDP-perosamine synthetase (Per) from *Escherichia coli* O157:H7 and synthesis of GDP-perosamine *in vitro*. Biochem. Biophys. Res. Commun. 363, 525–530 17888872 10.1016/j.bbrc.2007.08.184

